# Inhibitory Effects of Myrtucommuacetalone 1 (MCA-1) from *Myrtus Communis* on Inflammatory Response in Mouse Macrophages

**DOI:** 10.3390/molecules25010013

**Published:** 2019-12-18

**Authors:** Samreen Soomro, M. Ahmed Mesaik, Farzana Shaheen, Noureen Khan, Sobia Ahsan Halim, Zaheer Ul-Haq, Rafat Ali Siddiqui, Muhammad Iqbal Choudhary

**Affiliations:** 1 Dr Panjwani Center For Molecular Medicine and Drug Research, University of Karachi, Karachi 75270, Pakistan; mmesaik@hotmail.com (M.A.M.); sobia_halim@unizwa.edu.om (S.A.H.); zaheer_qasmi@hotmail.com (Z.U.-H.); rsiddiqui@vsu.edu (R.A.S.); hej@cyber.net (M.I.C.); 2 Faculty of Pharmacy, Northern Border University, Rafha 91911, Saudi Arabia; 3 Faculty of Medicine, University of Tabuk, Tabuk 71491, Saudi Arabia; 4 H. E. J. Research Institute of Chemistry, International Center for Chemical and Biological Sciences, University of Karachi, Karachi 75270, Pakistan; afnan.iccs@gmail.com (F.S.); noureenhej@gmail.com (N.K.); 5 Natural and Medical Sciences Research Center, University of Nizwa, P.O Box 33, Birkat Al Mauz, Nizwa, PC 616, Oman; 6 Food Chemistry and Nutrition Science, Agriculture Research Station, Virginia State University, Petersburg, VA 23806, USA

**Keywords:** respiratory burst, nitric oxide, inflammation, macrophages

## Abstract

(1) Introduction: Reactive oxygen species (ROS) and nitric oxide (NO) are key signaling molecules that play important roles in the progression of inflammatory disorders. The objective of this study was to explore the use of myrtucommuacetalone-1 (MCA-1), as a novel compound of natural origin and a potential anti-inflammatory agent. (2) Methodology: The anti-inflammatory potential of MCA-1, which was isolated from *Myrthus communis* Linn, was determined by assaying superoxide, hydrogen peroxide, and nitric oxide production in macrophages. Furthermore, the effects of the compound were analyzed via phosphorylation and translocation of the transcription factor NF kappa B, which is a key regulator of iNOS activation. The effect of MCA-1 on the inducible nitric oxide synthase (iNOS) enzyme was also examined using in silico docking studies. The anticancer potential for MCA-1 was evaluated with an MTT cytotoxic assay. (3) Results: In stimulated macrophages, MCA-1 inhibited superoxide production by 48%, hydrogen peroxide by 53%, and nitric oxide (NO) with an IC_50_ of <1 µg/mL. MCA-1 also showed a very strong binding pattern within the active site of the inducible nitric oxide synthase enzyme. Furthermore, 25 µg/mL of MCA-1 inhibited inducible nitric oxide synthase expression and abolished transcription factor (NFκB) phosphorylation and translocation to the nucleus. Cytotoxicity analyses of MCA-1 on 3T3 mouse fibroblasts, CC1 liver cell line, J774.2, macrophages and MDBK bovine kidney epithelial cell, yielded IC_50_ values of 6.53 ± 1.2, 4.6 ± 0.7, 5 ± 0.8, and 4.6 ± 0.7, µg/mL, respectively. (4) Conclusion: Our results suggest that MCA-1, a major phloroglucinol-type compound, shows strong anti-inflammatory activity and has a potential to be a leading therapeutic agent in the future.

## 1. Introduction

Regulation of inflammation is important because of its involvement in a number of serious chronic conditions, such as cancer, cardiovascular disease, and immune disorders. Lipopolysaccharide (LPS), the principal causative agent derived from the outer membrane of Gram-negative bacteria, regulates the release of inflammatory mediators associated with sepsis and other choric inflammatory disorders [1]. For example, LPS activates the nitric oxide (NO) pathway in macrophages [2]. When they encounter LPS, macrophages interact with toll like receptors (TLR 4) in association with CD14, which leads to the activation of cellular kinases, resulting in NFκB activation. This is the major inflammatory pathway that not only regulates NO release, but also is associated with the release of various cytokines during the inflammatory response [3]. Although the released NO helps to kill microbes, under chronic inflammation, however generation of free radicals if continues, are harmful and involve in many inflammation-associated pathologies. Under normal physiological conditions, basal NO is necessary for normal vascular function due to its effect as a neurotransmitter generated by neuronal and endothelial NO synthases. During inflammation, the inducible form of nitric oxide synthase becomes activated and causes NO generation, which in turn causes excessive vasodilation resulting in hypotension and septic shock. This may result in fatal complications in older and young people during bacterial infection, leading to sepsis [4]. One of the most toxic radicals derived from NO is peroxynitrite (ONOO), which is generated when NO couples with superoxide., which is released during phagocytosis. Generation of ONOO eventually reduces the bioavailability of basal NO and affects its endothelial relaxation function, thereby causing vasoconstriction and hypertension, which may lead to cardiovascular disease. On the other hand, excess formation of ONOO causes nitrosylation of proteins, which also contributes to cancer development [5,6]. In addition to this, NO also plays a role in heart and lung diseases, as well as in impotence [7,8]. The wide-ranging roles of NO in various pathological conditions prompted scientists to develop potent NO synthesis inhibitors [9]. We previously characterized the anti-inflammatory properties of myrtucommuacetalone 1 (MCA-1), a compound isolated from the traditional plant *Myrtus communis*. In the current study, we investigated the effects of myrtucommuacetalone 1 on NO induction via LPS-activated macrophages and further characterized its effects on downstream signaling pathways.

## 2. Results

### 2.1. Inhibition of NO Production by Myrtucommuacetalone 1 (MCA-1) in THP1 Monocyte and U138MG Cells

**Figure 1:**
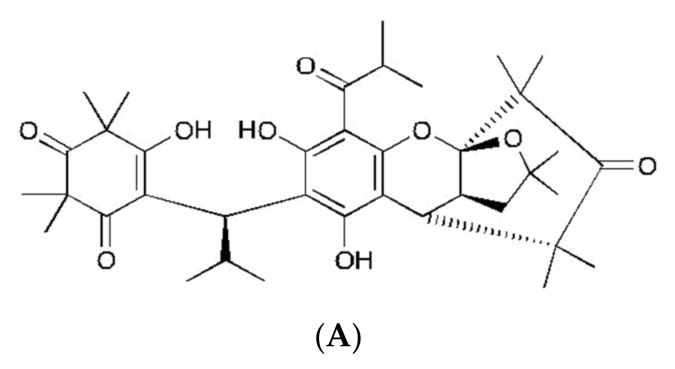

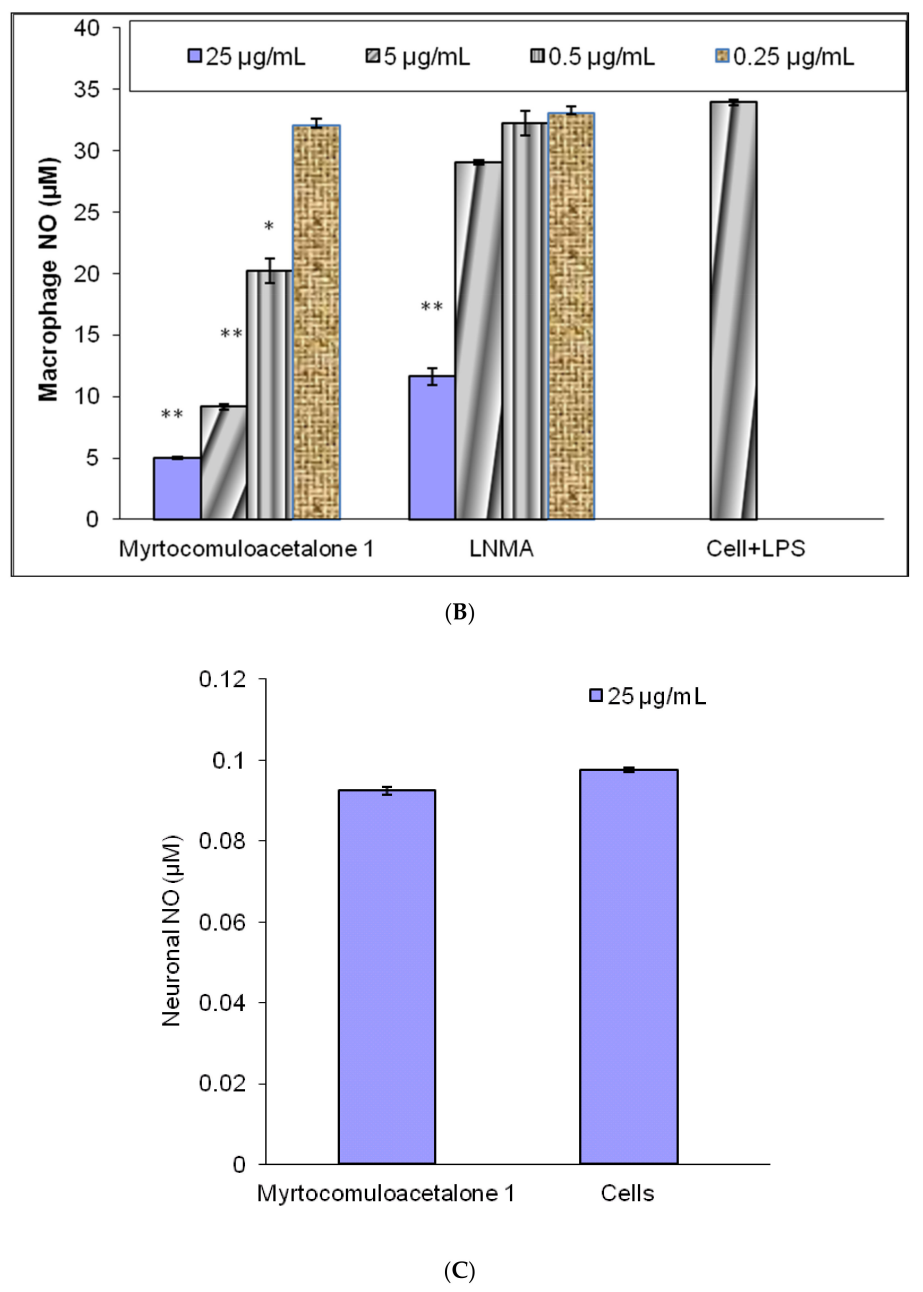
(**A**) Structure of myrtucommuacetalone 1 (MCA-1). (**B**) Effect of myrtucommuacetalone 1 (MCA-1) on nitric oxide (NO) release by activated THP1 monocytes. THP Cells (2 × 10^5^) were incubated in the presence (25, 5, 0.5, 0.25 µg/mL) or absence of the compound (control) for 48 h, and (**C**) the effects of the compound at 25 µg/mL on the basal level of neuronal NO in U 138MG neuronal cells were observed. The supernatant was analyzed for the presence of nitrite using the Griess method. Values are expressed as mean ± SD for triplicate experiments. The results were compared with N^G^ Monomethyl L arginine (LNMA), a known inhibitor of NO. * *p* < 0.05, ** *p* < 0.005.

The inhibitory effect of myrtucommuacetalone 1 was investigated using THP1 monocytes and U138MG cells to explore the effect of the compound on the inducible and constitutive forms of nitric oxide, respectively. The data presented in Figure 1A showed that MCA-1 exerted a dose-dependent inhibition of LPS-induced NO produced by THP1 cells (IC_50_ < 1 µg/mL), but showed no inhibitory effect on the basal level of NO produced by U138MG cells (Figure 1B).

### 2.2. Effect of MCA-1 on LPS-Induced NFκB and p38 Kinase Activation

**Figure 2:**
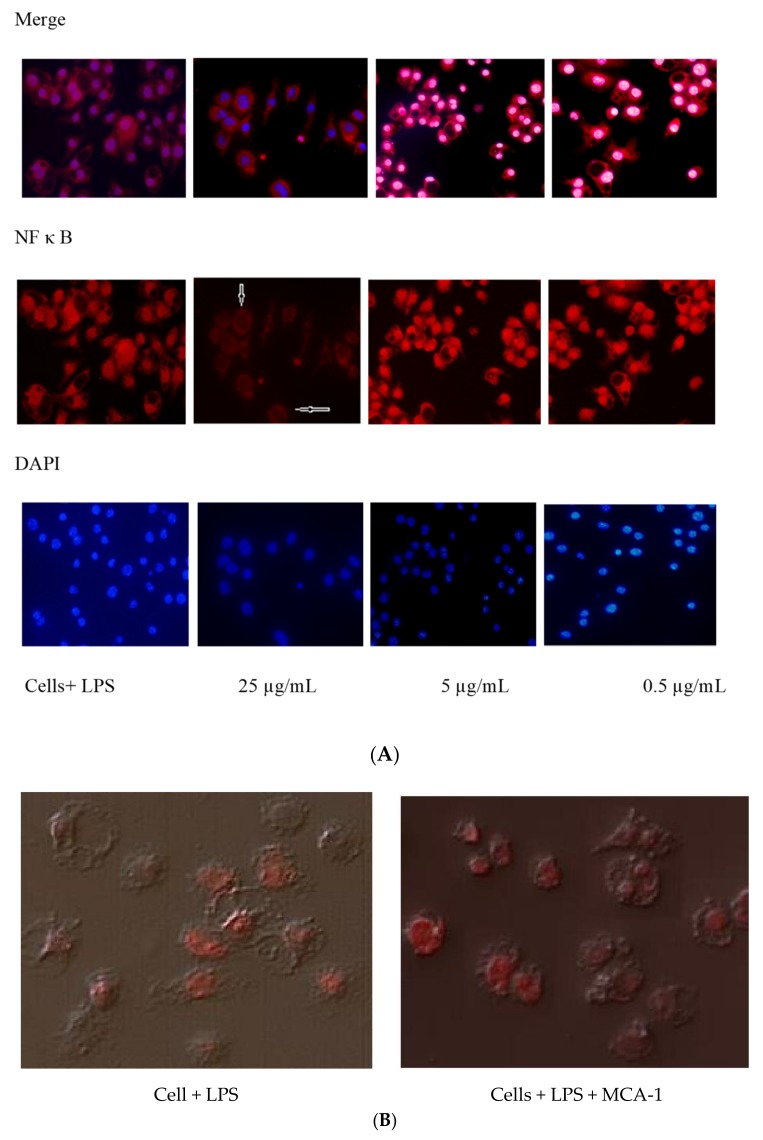
Lipopolysaccharide (LPS) induced nuclear translocation of the p65 subunit of NF kappa B and p38 kinase in J774 cells. The effects of MCA-1 at concentration 25 µg/mL (arrows) showed the absence of the NF kappa B transcription factor inside the nucleus at 25 µg/mL (**A**) and had no effect on p38 kinase translocation (**B**). The cells were examined at 20× magnification under the TRITC and DAPI channels using a Nikon TE-2000 epifluorescence microscope. The picture merge was performed using Adobe Photoshop.

NFκB and p38 kinase are transcription factors which are activated by inflammatory stimuli, such as LPS; the activation of these compounds is an essential step for the expression inflammatory genes, including iNOS. We investigated the mechanism of NO inhibition by the studied compound on the translocation of these transcription factors using immunocytochemistry techniques. The results showed that MCA-1 had no effect at concentrations of 5 and 0.5 µg/mL, but it almost completely abolished the NFκB translocation at 25 µg/mL (Figure 2). p38 remained unaffected at the highest concentration of this compound (Figure 2).

### 2.3. Effect of MCA-1 on iNOS Expression, NFκB Phosphorylation, and the iNOS Protein

**Figure 3:**
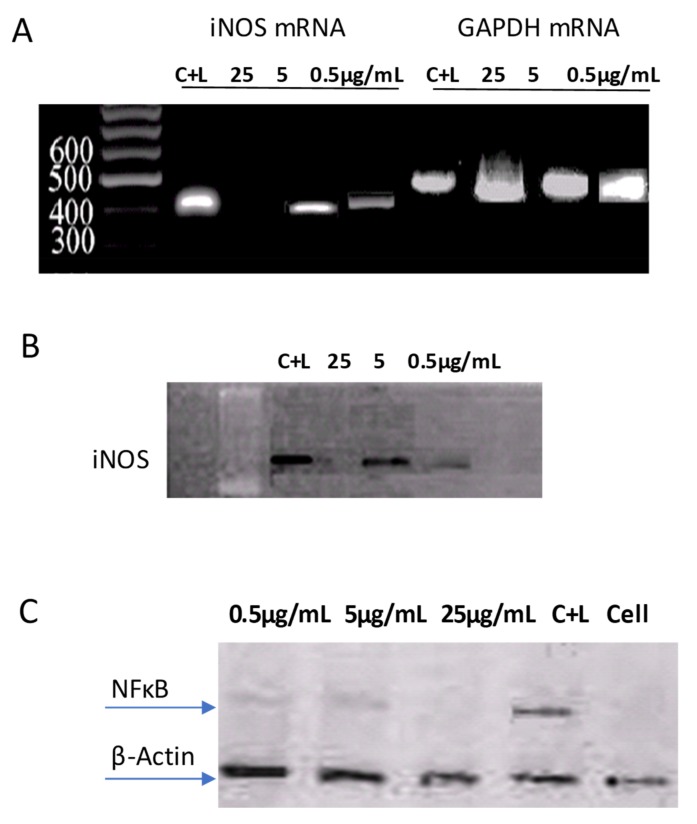

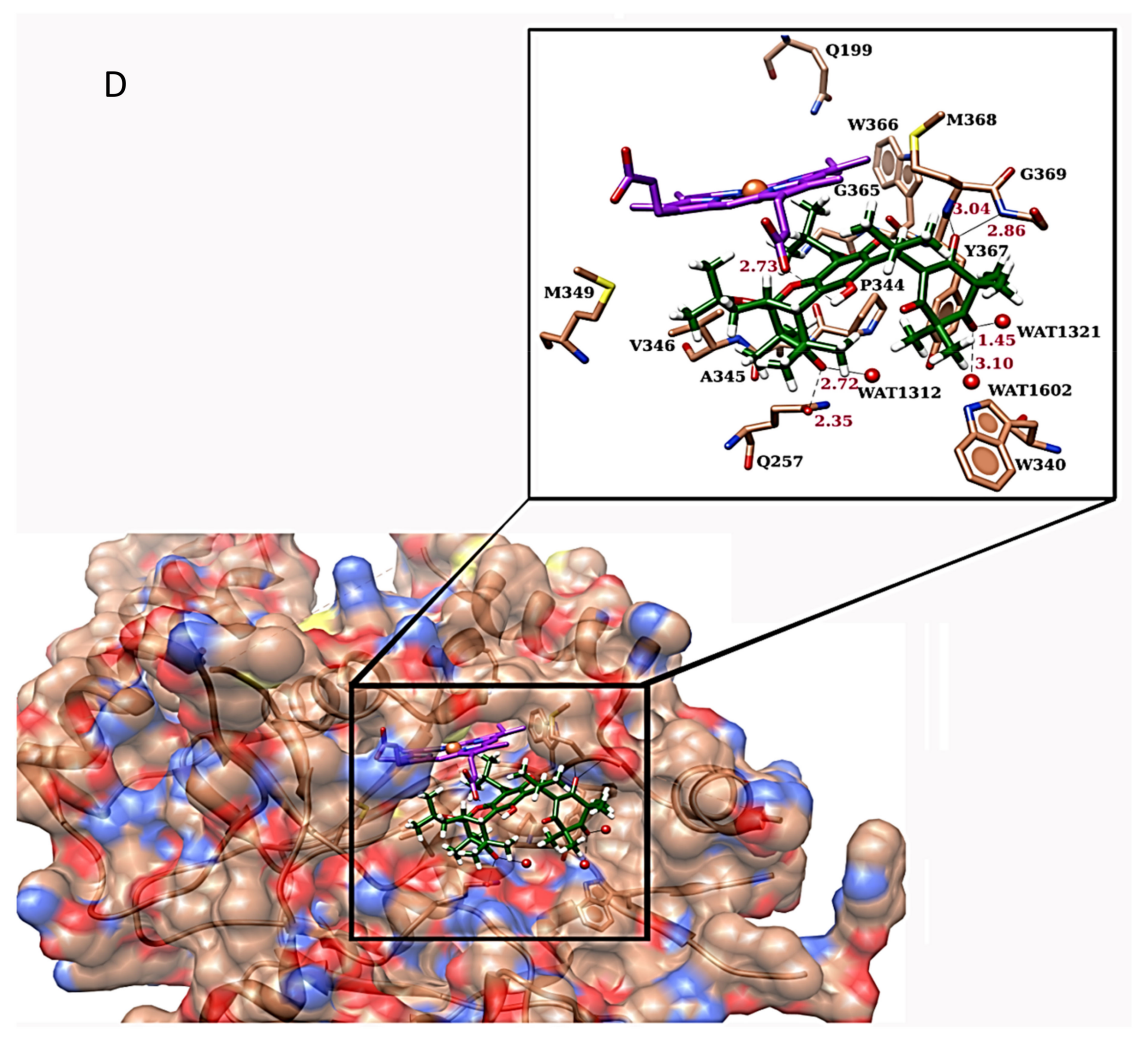
(**A**) Effect of MCA-1 on iNOS expression. J774 macrophages treated with 30 µg/mL of LPS to induce iNOS expression and total RNA were extracted to determine iNOS expression using RT-PCR, as described in the text. (**B**) Effect of MCA-1 on iNOS Protein (**C**)Effect of MCA-1 on NFkB phosphorylation. J774 macrophages were treated with LPS in the presence of varying concentrations of MCA-1 for 1 h to induce NFκB phosphorylation and for 48 h to monitor iNOS expression. A total of 35 μg of protein from the cell lysate was resolved on an SDS-PAGE and proteins were detected using specific antibodies against phosphorylated NFkB or iNOS, as described in the text. Sample loading was monitored by detecting β-actin with specific antibodies. The blots shown are representatives of two independent experiments. (**D**) The 3D structure of murine iNOS and the binding mode of MCA-1 at the active site of iNOS is shown in the box. The active site residues are depicted by the coral sticks, the active site of heme is shown by the purple sticks, and the compound is shown by the green sticks.

The effect of MCA-1 on mRNA expression of iNOS was determined using RT-PCR. The results shown in Figure 3A indicated that at 25 µg/mL, the compound significantly inhibited (*p* < 0.005) the expression of mRNA compared to the housekeeping gene GAPDH. Moreover, the quantification of iNOS expression by densitometry showed that MCA-1 at 25 µg/mL inhibited iNOS expression by 75% when compared to the control. LPS activated NFκB in macrophages, but it did not function as a transcription factor unless it was phosphorylated and translocated to the nucleus, where it was able to bind to DNA and transcribe genes, including for the enzyme iNOS. We therefore investigated the effect of MCA-1 on the phosphorylation of NFκB and the expression of the iNOS enzyme. The results indicated that MCA-1 inhibited the phosphorylation of NFκB (Figure 3B), and the expression of its downstream target, iNOS, was also inhibited at 25 µg/mL (Figure 3A,B).

### 2.4. Molecular Docking of iNOS Ligand with MCA-1

Docking simulations were performed to predict the binding mode of all of the iNOS ligands in the murine iNOS active sites and to evaluate their binding affinities.

The MCA-1 mediated several hydrogen bonding interactions with the surrounding active site residues, including Gly369, Gln257, the side-chain oxygen of heme, and three water molecules, i.e., WAT 1312, 1321, and 1602. A bidentate interaction was observed between the carbonyl oxygen at C15 of MCA-1 and the side-chain oxygen of Gln257 and WAT1312 at a distance of 2.35 Å and 2.72 Å, respectively. While the carbonyl oxygen at C3′ interacted with WAT 1321 and 1602 at a distance of 1.45 Å and 3.10 Å, respectively. The side-chain oxygen of heme contributed to the stabilization of the compounds by mediating hydrogen bonding with the hydroxyl group at the C7 position of MCA-1 (2.73 Å). Moreover, the compound was further stabilized by medicating bidentate interactions with the amide nitrogens of Gly369 and Met368 at a distance of 2.86 Å and 3.04 Å, respectively. Molecular interactions of the compound with the active site residues of iNOS are depicted in (Figure 3D).

In addition to molecular docking, the pharmacokinetic properties of MCA-1 were evaluated using in silico tools. The predicted profile suggested that the compound was noncarcinogenic, nonhepatotoxic, and nonmutagenic. Its predicted lethal dose (LD_50_) was very high (995 mg/kg), indicating that this compound was not lethal. Since the compound had a molecular weight, greater than 500 kDa, it violated one rule of Lipinski’s rules of five, which is applicable to oral drugs. However, this is common for naturally isolated compounds and drugs that target receptors. The lipophilicity, Caco-2 permeability, gastrointestinal absorption, and bioavailability measures were moderate. The compound showed no blood–brain barrier permeability. The skin permeation of the compound was also good. The compound may act as a P-glycoprotein substrate, while it does not act as inhibitor or substrate of cytochrome P450 CYP1A2, CYP2C19, CYP2C9, or CYP2D6; it may, however, inhibit CYP3A4. Moreover, the compound depicted no pan assay interference compounds (PAIN) alerts, suggesting it was safe. The cytotoxicity of different cancer cell line predictions showed that the could exhibit activity toward hepatoblastoma (HepG2), prostate carcinoma (DU-145), adult T-acute lymphoblastic leukemia (MT4), and melanoma (M19-MEL), while it did not show activity on nontumor cell lines. These data are tabulated in Table 1.

### 2.5. Effect of MCA-1 on Cell Viability and Anticancer Activity

**Figure 4:**
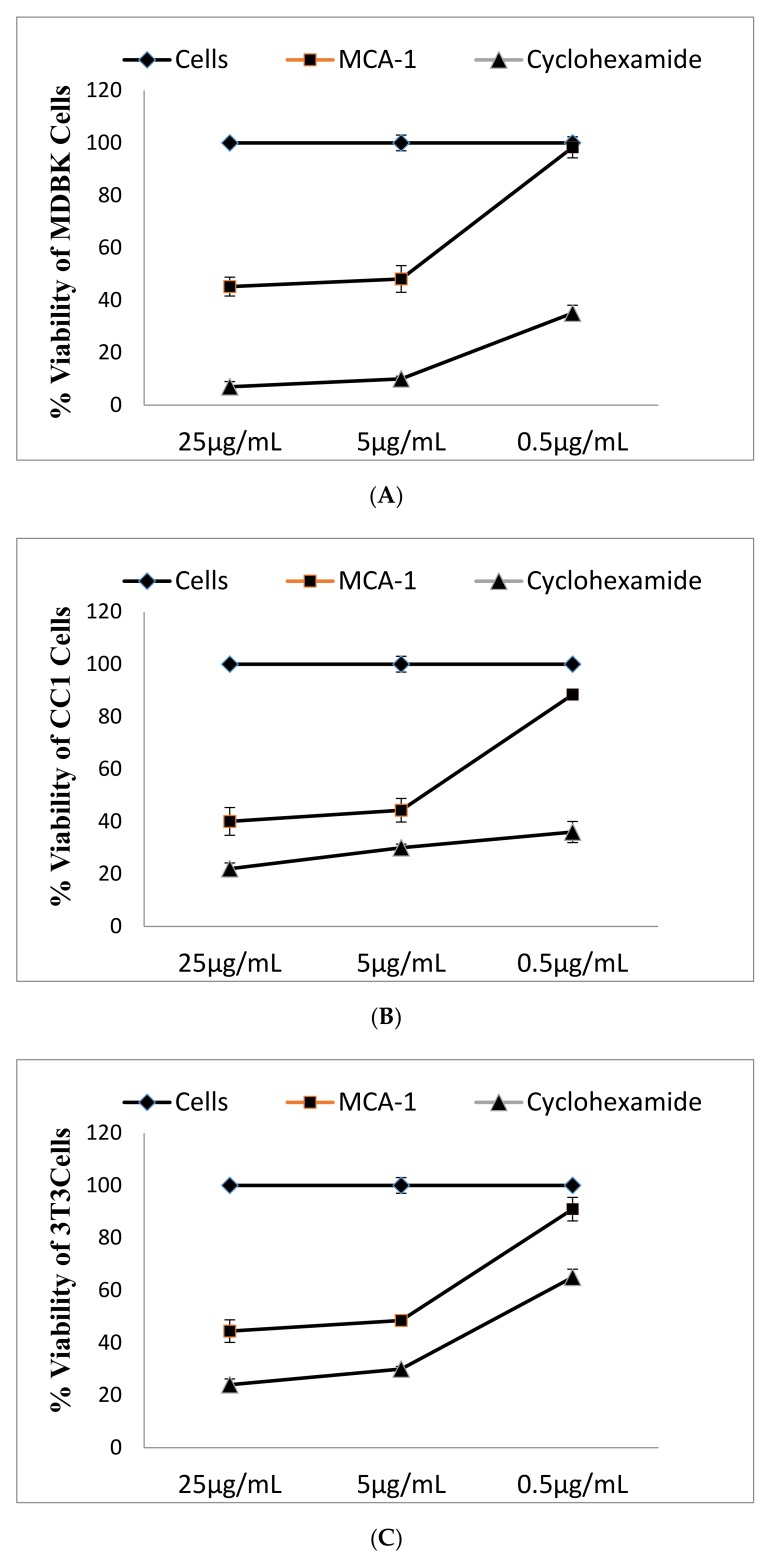

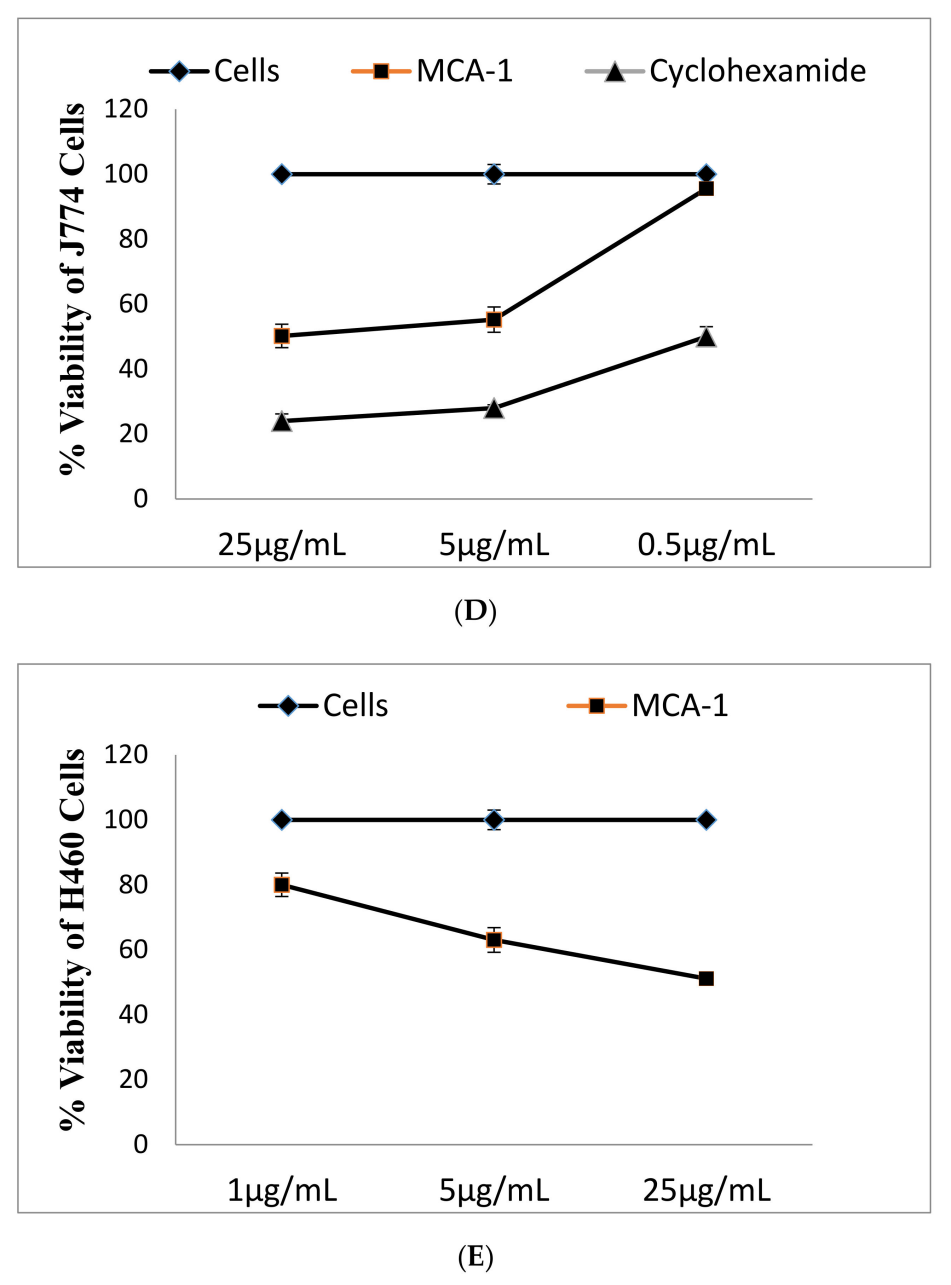
Effect of MCA-1 on cellular toxicity. MDBK kidney (**A**), CC1 liver (**B**), 3T3 NIH mouse fibroblasts (**C**) and J774.2 mouse macrophage (**D**) cell lines were incubated in the absence (■) and presence (♦) of varying concentrations of MCA-1 for 48 h. The cytotoxic effects of MCA-1 were determined using the MTT assay, as described in the text. The effect of the cytotoxic drug cyclohexamide (▲) was also determined for comparison. (**E**) Anticancer activity of myrtocomuloaetalone1 on the H640 lung cancer cell line. H640 cells were incubated in the presence (■) or absence (♦) of varying concentrations of MCA-1 for 48 h. The cell viability was determined by FACS analysis, as described in the text. The values are represented as the mean ± SD for triplicate experiments. The data were analyzed by cell quest pro. Significant differences are reported at *p* < 0.05. The values are represented by the mean ± SD for triplicate experiments.

The effect of MCA-1 on cell viability was determined in MDBK, CC1, J774 3T3 fibroblast, and lung cancer cells, and the IC_50_ for each line was calculated. The data are presented in Figure 4A–E. The IC_50_ of MCA-1 on these cells when incubated for 48 h appeared to be within the 4–6 µg/mL range (MDBK kidney cells, 4.5 ± 1.1; CC1 liver cells, 4.6 ± 0.7; 3T3NIH mouse fibroblasts, 6.53 ± 1.2; J774.2 macrophages, 5 ± 0.8 µg/mL). These compounds appeared to be far less toxic compared to the Cyclohexamide, which showed an IC_50_ on MDBK kidney cells of 1.2 ± 0.4, CC1 liver cells of 0.02 ± 0.001, 3T3 mouse fibroblast cells of 0.13 µg/mL, and macrophages of 0.2 ± 0.9 µg/mL. 

MCA-1 was analyzed for its anticancer activity on H460 lung cancer cells. The data presented in Figure 4E indicated that MCA-1 had a dose-dependent effect on cell viability; at 25 µg/mL the compound inhibited cell viability up to 47% with an IC_50_ value of 34.4 ± 0.9 compared to cisplatin, which had an IC_50_ of 5.7 ± 0.3 μg/mL, showing that the compound was moderately effective against lung cancer cells.

### 2.6. Effect of Compound on ROS Generation and H_2_O_2_ Production

The effects of MCA-1 were determined with respect to free radical formation during an oxidative burst. Data (not shown) indicate that MCA-1 at 25 μg/mL had no effect on hypochlorous acid (HOCl) production, which is generated via a myeloperoxidase-dependent pathway, whereas it had a significant inhibitory effect (42.5%) on superoxide generation, which is produced via NADPH oxidase. In addition to this, MCA-1 also inhibited hydrogen peroxide by 53%, which is produced due to the activity of superoxide dismutase (Table 2).

## 3. Discussion

Nitric oxide is produced in response to LPS, which initiates the downstream signaling cascade and activates transcription factors, including NFκB, ERK, P38 kinase, and AP1 [10]. The activation of these transcriptional factors causes gene expression for the synthesis and release of inflammatory cytokines and reactive oxygen species. We determined the effects of MCA-1 on the activation of transcription factors NFκB and p38 kinase in this investigation. Our data, as described in Figure 2, indicated that MCA-1 completely inhibited NFκB activation at 25 µg/mL, but did not affect p38 kinase, suggesting that the anti- inflammatory effects of MCA-1 are not related to LPS-receptor binding or upstream signaling involved in p38 activation. These effects appeared to be specific to NO-mediated NFκB activation. Inhibition of NFκB activation was shown to cause a reduction in the expression of iNOS mRNA [11]. Consistent with these observations, our results indicated that MCA-1, besides inhibiting the expression of iNOS mRNA, inhibited the translation of iNOS protein at a concentration of 25 µg/mL concentration. Previous studies also demonstrated the significance of NFκB in inflammatory disease through loss-of-function approaches. This study revealed that NFκB induced catabolic gene expression through NFκB response elements, thereby promoting the expression of major pro-inflammatory and destructive mediators, including cyclooxygenase 2 (COX2), prostaglandin E2 (PGE2), and inducible nitric oxide synthase (iNOS). Particularly, the loss of iNOS appeared to attenuate the process of inflammation [12,13,14]. Interestingly, the data shown in Figure 1A above indicated that LPS-induced NO production in vitro was inhibited in a dose-dependent manner. We therefore performed in silico inducible nitric oxide synthase enzyme (iNOS) molecular docking studies with respect to MCA-1 via the Freud docking method. Our results showed that the compound had a very strong binding pattern within the active site of enzyme. This observation strongly suggests that MCA-1 is a strong inhibitor of the inducible nitric oxide synthase enzyme. To our knowledge, this is the first study to show MCA-1 as a novel, potent anti-inflammatory agent which mediates its effects through inhibition of NFκB activation and iNOS enzyme inhibition.

During the oxidative burst mechanism, reactive oxygen species (ROS), such as O_2_^−^, H_2_O_2_, and OH^.^ are generated via normal cellular processes. Cellular antioxidant enzymes and free-radical scavengers normally protect cells from the toxic effects of ROS. However, when ROS generation exceeds the cellular antioxidant system of the cells, oxidative damage of the cellular macromolecules, including lipids, proteins, and nucleic acids, occurs. If continued, this leads to various pathological conditions and cell death due to cellular damage. [15] The results of the current study indicated that MCA-1 had a moderate inhibitory effect on reactive oxygen species produced during inflammation, including superoxide generation, which is produced through NADPH oxidase and hydrogen peroxide generated by the activity of superoxide dismutase. However, it did not exert any significant effect on hypochlorous acid (HOCl) production, which is generated via a myeloperoxidase-dependent pathway. Furthermore, MCA-1 was found to have about 10-fold less cellular toxicity than cyclohexamide when tested on J774 macrophages, 3T3 fibroblasts, CC1 liver cells, and MDBK kidney cells. According to in silico pharmacokinetic studies, the lethal dose is high (995 mg/kg), indicating that this compound is not lethal. The compound has a molecular weight of more than 500 kDa, which is common for naturally isolated compounds and drugs that target receptors. Therefore, this compound has a potent utility to become a safe anti-inflammatory agent.

Chronic inflammation has a strong association with the development and progression of cancer, especially in patients with chronic inflammatory pulmonary disease. This may lead to lung cancer due to exaggerated inflammatory mediators [16]. We therefore investigated the effects of MCA-1 with respect to its anticancer potential using H460 lung cancer cells. Our data, as presented in Figure 4, indicated that MCA-1 also had moderate anticancer activity for H460 lung cancer cells. Furthermore, predicted values for cytotoxicity on different cancer cell lines showed that the compound provided activity toward hepatoblastoma (HepG2), prostate carcinoma (DU-145), adult T-acute lymphoblastic leukemia (MT4), and melanoma (M19-MEL), with no activity toward nontumor cell lines. Nevertheless, further research is required to explore the anticancer effects of MCA-1 against other cancer cell lines of lungs and other tissues in vitro.

The physicochemical and pharmacokinetic profiles of MCA-1 predicted that it was not carcinogenic or mutagenic. The lipophilicity, CaCO_2_ permeability, gastrointestinal absorption, and bioavailability measures were all moderate. The compound showed no blood–brain barrier permeability, but the skin permeation was good. The compound may act as a P-glycoprotein substrate, but it did not act as inhibitor or substrate of cytochrome P450, CYP1A2, CYP2C19, CYP2C9, and CYP2D6. However, it may inhibit CYP3A4, which metabolizes most medications. Moreover, the compound showed no PAIN alerts, suggesting it is safe and the compound is not a nonspecific inhibitor.

## 4. Materials and Methods

### 4.1. Plant Material, Extraction and Isolation

Methanolic extract from leaves of *Myrtus communis* Linn was used for further purification and isolation of the pure compound. The repeated chromatographic separation yielded a new compound, myrtucommuacetalone 1 (MCA-1) (Figure 1A). The details of myrtucommuacetalone 1 isolation, purification, and structural chemistry were described previously [2].

### 4.2. Animals, Cells, Chemicals, and Reagents

Balb/C mice were maintained in the institute’s animal facility under a pathogen-free environment. Experiments were performed under the ethical guidelines of international association for study and pain in conscious animals and guidelines were set by scientific advisory committee animal care, use and standard international center for chemical and biological sciences (Protocol No ASP2016-0045). Balb/C monocyte macrophage J774.2, 3T3 mouse fibroblasts, MDBK kidney cells, THP1 monocytes, and U138MG cells were obtained from the European Collection of Cell Cultures (Salisbury, UK). Delbuco Modified Eagle Medium (DMEM), Minimum Essential Media (MEM), mercaptoethanol, sodium dodecyl sulphate (SDS), isopropyl alcohol, chloroform, sodium hydrogen carbonate, and Vectashield anti-fade mountant were obtained from Sigma-Aldrich (Steinheim, Germany). Fetal bovine serum (FBS) and streptomycin/penicillin were purchased from GIBCO BRL (New York, NY, USA). The chamber slide was obtained from Nalge Nunc International (New York, NY, USA). Flasks with a volume of 75cc and multiwall (96, 48, 6) plates with transparent bottoms, were obtained from Corning (New York, NY, USA). *Escherichia coli* lipopolysaccharide (LPS) was obtained from DIFCO Laboratories (New York, MI, USA). Phosphoric acid, naphthyl-ethylene diamine dihydrochloride, sulfoniamide, sodium nitrite, bromophenol blue, and ammonium persulfate were obtained from Fisher Scientific (Leicestershire, UK) and RIPA lysis buffer was obtained from Millipore (New York, NY, USA). Prestained protein molecular weight markers were obtained from Thermo Scientific (Rockford, IL, USA). Bisacrylamide was obtained from BioM laboratories, (Cerritos, CA, USA). Tween-20, tetramethylethylenediamine (TEMED), glycerol, and acrylamide were purchased from Merck (KGaA, Germany). Sodium azide and sodium chloride were obtained from Wako, (Shanghi, China). Horse reddish peroxidase, HRP-substrate and Immuno-star Western C kit were obtained from BioRad laboratories (New York, NY, USA). Tris-HCl was obtained from Vivantis biochemical (New York, NY, USA). Horseradish peroxidase and scopoletin were obtained from TCI (Tokyo, Japan). 2,7-dichlorofluorescein and nitrotetrazolium blue were obtained from Bio Basic (New York, NY, USA). Dimethylsulfoxide, L-glutamine, PMA phorbol myristate 13 acetate, and ethanol were obtained from MP Biomedical (Ohio, OH, USA). Propedium iodide was from Fluka (Rochester, NY, USA). Trizole and a Superscript Reverse Transcriptase (RT) Kit were purchased from Invitrogen (Burlington, Canada). Secondary fluorescent label goat anti-rabbit antibody was obtained from Sigma, (Poole, UK), goat anti-rabbit HRP conjugated polyclonal antibody was purchased from Santa Cruz Biotechnology (Santa Cruz, CA, USA), and rabbit polyclonal antibodies for iNOS, the p65 subunit of phosphorylated NFκB, and p38 were obtained from Abcam (Farmingdale, NY, USA).

### 4.3. Cell Culture

J774.2 mouse macrophages, 3T3 mouse fibroblasts, MDBK kidney cells, U138MG neuronal cells, and THP1 monocyte cells were maintained at sub-confluence in DMEM medium supplemented with 10% FBS, whereas the CC1 liver cell line was grown in MEM with 12% FBS. For anticancer activity, the H460 lung cancer cell line was maintained in phenol red free RPMI medium supplemented with 10% FBS, 1% penicillin/streptomycin (Pen/Strep), and 1% glutamine. Cell lines were maintained in a humidified incubator at 37 °C with 5% CO_2_.

### 4.4. Determination of Nitrite Oxide Concentration

The human THP1 monocytes were differentiated into macrophages by incubating with 20 ng/mL of PMA for 16–18 h. Flasks were kept at 37 °C in humidified air containing 5% CO_2_. Cells (10^6^ cells/mL) were then transferred to a 96-well plate. Nitric oxide synthase-2 (NOS-2) in THP1 cells was induced by 30 µg/mL *E. coli* LPS, whereas neuronal cells were kept deprived of FBS for 24 h to generate NO. The cell culture supernatant was collected after 48 h and the nitrite concentration was measured in the presence or absence of the test compounds using the Griess method, as described earlier [17,18]. In brief, 50 µL of 1% sulphanilamide in 2.5% phosphoric acid and 50 µL of 0.1% naphthyl-ethylene diamine dihydrochloride in 2.5% phosphoric acid were added to 50 µL of culture medium. After 10 min of incubation at room temperature, the absorbance was read at 550 nm. The concentrations of nitrite (μM) were calculated from a standard curve using sodium nitrite as a reference compound.

### 4.5. Determination of NFκB and p38 Kinase Translocation

J774.2 mouse macrophages were plated in a chamber slide (10,000 cells/well) along with 10 µg/mL of LPS and 25 µg/mL of the test compound. The slides were incubated for 1 h at 37 °C in 5% CO_2_ environment. The cells were fixed using 4% paraformaldehyde and then permeabilized using Triton X-100. The fixed cells were blocked by incubating with 10% FBS for 1 h at room temperature. Cells were washed once and then incubated with primary antibody (p65 subunit of NFκB, diluted to 1:50 in medium containing 10% FBS) overnight at 4 °C. The expression of NFκB was detected using secondary fluorescent label goat anti-rabbit antibody (1:200 in PBS containing 1% FBS) for 90 min at room temperature. Subsequently, the slides were washed thrice in PBS before applying Hoechst 33342 stain (1:1000 dilution in PBS) for 15 s and mounting the coverslips using Vectashield anti-fade mountant [18,19]. Images were acquired using fluorescence microscopy and the results were recorded by manual counting of cells for p65 translocation into the nucleus in three random areas per slide.

### 4.6. Determination of iNOS Expression by RT-PCR

J774.2 cells (10^6^/well) were plated in 6-well plates and then activated with LPS (20 µg/mL) in the absence or presence of the test compounds (25 µg/mL). Our preliminary data indicated that iNOS mRNA reached its maximum level by 6 h and declined after 24 h. We therefore collected the cells for mRNA isolation after 6 h of stimulation. Briefly, cells were rinsed with ice cold PBS once and then lysed directly in a culture dish by adding trizol reagent. The mRNA was isolated according to manufacturer’s instructions. cDNA was synthesized with 1 µg of mRNA using the Superscript RT Kit and amplified using specific primers corresponding to mouse iNOS, whereas Glyceraldehyde 3-phosphate dehydrogenase (GAPDH) primers were used as the internal standard. The primer sequences for iNOS were the sense primer 5′CCC TTC CGA AGT TTC TGG CAG3′ and the antisense primer 5′GGC TGT CAG AGC CTC GTG GCT3′, whereas the primers for GAPDH were the sense primer 5′GAA GGG CTA ATG ACC ACA GTC3′ and the antisense primer 5′TAG CCA TAT TCG TTG TCG ATC3′. The calculated annealing temperatures for iNOS and GAPDH were 60 °C and 58 °C, respectively.

The PCR reaction was set to start at 65 °C for 2 min, then undergo denaturation at 94 °C for 1 min, annealing at 60 and 58 °C for 1 min, then extension at 72 °C for 2 min. This reaction was repeated for 30 cycles. At the end of 30th cycle, a final end-extension for 10 min at 72 °C was performed and the PCR product was resolved on 1% agarose gel.

### 4.7. Detection of iNOS and p65 Proteins

After the desired incubation time, cell lysates were prepared as described earlier. The Bradford Lowry method was used to measure the protein content of the samples. Immunoblot analyses for p65 and iNOS were carried out, as described earlier [17,18,19,20,21]. Briefly, protein samples (30 μg) were separated by SDS-PAGE on a 10% polyacrylamide gel and the resolved proteins were transferred to a nitrocellulose membrane. The expressions of specific proteins were detected with rabbit polyclonal antibodies for iNOS, the p65 subunit of phosphorylated NFκB, and p38 using goat anti-rabbit HRP conjugated polyclonal antibody. The bands were visualized using a chemiluminescent substrate via the Versa Doc imaging system (Innotech, Cridersville, OH, USA).

### 4.8. Determination of Superoxide Production by NBT Assay

For the induction of inflammatory responses, mice were injected intraperitoneally (ip) with 1 mL FBS, and inflammatory peritoneal macrophages were obtained from the animals three days later. This procedure was performed in a laminar flow chamber to ensure sterile conditions, as described previously [22]. The peritoneal macrophages (1 × 10^7^/100 µL) were suspended in the RPMI-1640 media, supplemented with 10% heat-inactivated FBS, and seeded in 96-well cell culture plates to measure NADPH oxidase activity using a modified colorimetric nitro blue tetrazolium (NBT) assay, as previously described [23]. Cells were incubated with the test compounds for 30 min at 37 °C, then 100 µL of NBT solution (1 mg/mL) and 50 µL of phorbol-12-myristate-13-acetate (PMA, 10 µg/mL) were added. The cells were incubated again under the same conditions for another 90 min. Diphenyleneiodonium was used as a negative control. The blue color formazan that formed due to NADPH activity was extracted with organic solvent (DMSO), and the absorbance was read at 570 nm. The control was run simultaneously using similar conditions without adding the test compounds. The percentage inhibition of NBT reduction was calculated using Equation (1).

(1) $$\%\mathrm{inhibition}=100-\frac{\left[\left(\mathrm{OD}\;\mathrm{of}\;\mathrm{sample}\;\mathrm{treated}\;\mathrm{cells}-\mathrm{OD}\;\mathrm{of}\;\mathrm{cells}\;\mathrm{alone}\right)\right]}{\left[\left(\mathrm{OD}\;\mathrm{of}\;\mathrm{PMA}\;\mathrm{treated}\;\mathrm{Cells}-\mathrm{OD}\;\mathrm{of}\;\mathrm{cells}\;\mathrm{alone}\right)\right]}\times 100\%$$

### 4.9. H_2_O_2_ Determination

Mouse macrophages J774.2 (2 × 10^5^ cells /mL in HBSS) were seeded in 24-well plates (Corning, New York, NY, USA) in the presence of 1 mM NaN_3_ as myeloperoxidase (MPO) inhibitor [24]. Cells were then incubated with the test compound (25 µg/mL) and activated with PMA (500 ng/mL) in a CO_2_ incubator at 37 °C for 2 h. After incubation, the medium was removed and the cells were washed gently once, then carefully lysed using ice-cold 1% triton X-100. A total volume of 100 μL of freshly prepared reaction mixture (50 µM scopoletin and 1 U/mL HRP) was added into the transparent-bottomed, solid-edged plate, and then 30 µL of cell lysate was added to the wells. The reaction plate was incubated for 40 min under shaking conditions at 37 °C. H_2_O_2_ secreted by cells oxidized the scopoletin to a nonfluorescent state in a reaction catalyzed by horseradish peroxidase. The plate was kept on ice to stop the reaction and the decrease in fluorescence was measured using a fluorimeter (CHAMELEON^TM^V multilabel microplate reader, Turku, Finland) at excitation and emission wavelengths of 355 nm and 460 nm, respectively. The results were quantified using an external standard curve based on defined concentrations of H_2_O_2_.

### 4.10. Determination of Cytotoxicity

The cytotoxic effect of myrtucommuacetalone 1 on various cell lines was determined with an MTT assay [25]. MDBK cells (1 × 10^5^ cells/mL), CC1, and 3T3 (6 × 10^4^) cells were plated in 96-well flat-bottomed plates for 24 h to allow cell attachment. The media was replaced and various concentrations of the test compounds (0.5, 5, 25 µg/mL) were added into the wells; the plates were further incubated for 48 h. The medium was removed by flipping the plate. MTT solution (200 µL) was added to the wells and the plates were incubated for 4 h at 37 °C. The solutions were aspirated from each well and the cells were mixed with 100 µL of DMSO for 15 min on a rocker to dissolve the formazan crystals. The absorbance was measured at 570 nm using the microplate Spectra Max 340 (Molecular Devices, Silicon Valley, CA, USA) and the IC_50_ values were calculated. Cyclohexamide was used as a positive control to induce cellular toxicity.

The anticancer activity of the test compound was determined by measuring viable/dead cells using flow cytometry. H460 lung cancer cells (2 × 10^5^ cells /well) in 200 µL volume were added to a 24-well plate (corning, New York, NY, USA). The test compounds (0.5, 5, 25 µg/mL) were added to these cells and the plates were incubated for 48 h. Cells were harvested after incubation and processed for FACS analysis. Briefly, the cells and the supernatant were collected and washed 3 times using cell culture medium at 2500 rpm for 10 min. Finally, the cell pellets were suspended in 200 µL of PI buffer (0.2% BSA and 0.1% NaN_3_ in PBS) and incubated on ice. Before FACS (FACS caliber, Becton Dickinson San Jose, CA, USA) analysis, propidum iodide (0.5 mg/mL) was added to the samples and the shift in the peak between the labeled and unlabeled cells was recorded. Cisplatin was added as a control instead of the compound. Data were analyzed using cell quest pro and the graph was plotted using the concentration of the compound against the percentage of live cells on MS Excel.

### 4.11. Molecular Docking Studies for iNOS Inhibition

Molecular docking studies were conducted to understand the binding mechanism of iNOS inhibitors using the FRED docking program. All of the test compounds were docked in the binding pocket of murine iNOS (PDB code: 2ORT). Prior to the docking experiment, water molecules (except WAT1312, WAT1321, and WAT1602, which play important roles in protein–ligand interactions) were removed from the receptor file, and hydrogen was added. The heme group and the co-crystallized ligand [(3*S*)-1-(1,3-benzodioxol-5-yl-methyl)-3-[4-(1*H*-imidazol-1-yl)phen-oxy]piperidine)] were retained in order to define the active site.

#### 4.11.1. FRED 3.0.0

FRED is a fast and effective docking application that performs rigid docking. FRED performs a systematic, exhaustive, nonstochastic examination of all possible poses within the protein active site and filters for shape complementarity [26] and pharmacophoric features before selecting and optimizing poses using the Chemgauss4 scoring function. FRED requires a prepared receptor file and a ligand conformer library. The multi-conformer libraries of each ligand were generated by OMEGA [27]. A dielectric constant of 4.0 and the MMFF94s force field were chosen. The receptor files were prepared using the FRED receptor setup utility with the supplied protein structure in combination with a shape-based site detection algorithm and the position of a known bound ligand [(3*S*)-1-(1,3-benzodioxol-5-yl-methyl)-3-[4-(1*H*-imidazol-1-yl)phenoxy]piperidine)]. Contours were created to limit the search space to the volume enclosed by bound ligand. The Chemgauss4 scoring function was used to rank the compounds. Fifty docking poses were saved for analysis.

#### 4.11.2. Redocking Experiment

In order to evaluate the effectiveness of the FRED docking program and to establish the parameters to be used in the docking calculations, redocking calculations were performed using the crystallographic complex obtained from the PDB. The complex was 2ORT [28], which is a structure of murine inducible nitric oxide synthase (iNOS) complexed with [(3*S*)-1-(1,3-benzodioxol-5-yl-methyl)-3-[4-(1*H*-imidazol-1-yl)phenoxy]piperidine)] with a resolution of 1.87 Å. The reference ligand was extracted from the protein file, the atom and bond types were rectified, protons were added, and a database of 30 conformations was generated by OMEGA. The reference ligand was redocked into the active site of iNOS and the resulting geometry was compared with the X-ray crystal structure. The FRED predicted a geometry (figure not shown) that was superimposed with the X-ray structure of the reference ligand. The root-mean-square deviation (RMSD) of these two conformations was 0.341 Å, indicating good agreement with the crystal.

### 4.12. Physicochemical and Pharmacokinetic Predictions of MCA-1

The physicochemical and pharmacokinetic profiles of MCA-1 were predicted by SwissADME (http://www.swissadme.ch/index.php) and PreADMET (https://preadmet.bmdrc.kr/). The toxicity of the compound was evaluated by ProTox II (http://tox.charite.de/). The cell-line cytotoxicity was predicted by CLC-Pred (http://way2drug.com/Cell-line/)

### 4.13. Data Analysis

The results were expressed as the mean ± standard deviation (SD). Analysis of variance (ANOVA) was used for the statistical analysis of multiple comparisons of data. *P*-values of less than 0.05 were considered statistically significant (* *p* < 0.05, ** *p* < 0.005).

## 5. Conclusions

In conclusion, the present study demonstrated that MCA-1, isolated from *Myrtus communis* Linn and a phloroglucinol derivative, is an elective and potential inhibitor of NO synthesis during the oxidative burst by macrophages in vitro at a concentration of <1 µg/mL. Its effects were mediated via suppressing the activation of the transcription factor NFκB by inhibiting its phosphorylation and translocation, but not through activation of p38 kinase. The compound significantly suppressed the iNOS gene and protein expression as shown in vitro and exhibited a strong binding pattern in the binding site of iNOS enzyme in silico. The compound also demonstrated moderate anticancer activity against lung cancer cells in vitro. In contrast, toxicity to normal cell lines of the kidney, fibroblast, macrophage, and liver was observed at a concentration greater than its potential activity for nitric oxide inhibition. The results suggest that this compound could potentially be a leading entity in anti-inflammatory drug discovery research, and could be beneficial to target NO-mediated pathologies at a low concentration without causing toxic effects.

## Figures and Tables

**Table 1:** Predicted ADMET properties of MCA-1.

**Physicochemical Properties**	Formula: C38H52O9; molecular weight = 652.81 g/mol; number of heavy atoms = 47, volume = 617.87; number of aromatic heavy atoms = 6; fraction Csp3 = 0.68; number of rotatable bonds = 5; number of H-bond acceptors = 9; number of H-bond donors = 3; molar refractivity = 180.26; TPSA = 147.43 Å²; lipophilicity [Log *P*_o/w_ (iLOGP)] = 3.22; water solubility (Log *S*) = −8.05; Caco2 (human colorectal carcinoma cell permeability) = 22.8225 nm/sec
**Pharmacokinetic Properties**	GI absorption = moderate; BBB permeant = no; P-gp substrate = yes; CYP1A2 inhibitor = no; CYP2C19 inhibitor = no; CYP2C9 inhibitor = no; CYP2D6 inhibitor = no; CYP3A4 inhibitor = yes; Log *K*_p_ (skin permeation) = −5.33 cm/s
**Drug Likeness**	Lipinski = yes, 1 violation: MW > 500; Ghose = no, 4 violations: MW > 480, WLOGP > 5.6, MR > 130, number of atoms > 70; Veber = no, 1 violation: TPSA > 140; Egan = no, 2 violations: WLOGP > 5.88, TPSA > 131.6; Muegge = no, 2 violations: MW > 600, XLOGP3 > 5; bioavailability score = 0.56
**Medicinal Chemistry**	PAINS = 0 alert; Brenk = 1 alert: beta_keto_anhydride; leadlikeness = no, 2 violations: MW > 350, XLOGP3 > 3.5; synthetic accessibility = 7.31
**Toxicity**	AMES test = nonmutagenic; carcinogenicity = none; predicted LD_50_ = 995 mg/kg; hepatotoxicity = none
**Cancer Cell line Prediction**	Hepatoblastoma (HepG2), prostate carcinoma (DU-145), adult T-acute lymphoblastic leukemia (MT4), and melanoma (M19-MEL)
**Nontumor Cell Line Prediction**	None

**Table 2:** Effects of myrtocomuloacetalone1 on reactive oxygen species. Results are expressed as the mean value of three readings.

Compound	ROS (IC_50_) µg/mL	Oxidative Burst% Inhibition at 25 µg/mL	
		O_2_^−^	H_2_O_2_
MCA-1	>100	42.5	53
